# Switching Patients with Non-Dialysis Chronic Kidney Disease from Oral Iron to Intravenous Ferric Carboxymaltose: Effects on Erythropoiesis-Stimulating Agent Requirements, Costs, Hemoglobin and Iron Status

**DOI:** 10.1371/journal.pone.0125528

**Published:** 2015-04-30

**Authors:** Jorge Eduardo Toblli, Federico Di Gennaro

**Affiliations:** Department of Nephrology, Hospital Alemán, Buenos Aires, Argentina; University Jean MONNET of SAINT-ETIENNE, UNITED STATES

## Abstract

**Background:**

Patients with non-dialysis-dependent chronic kidney disease (ND-CKD) often receive an erythropoiesis-stimulating agent (ESA) and oral iron treatment. This study evaluated whether a switch from oral iron to intravenous ferric carboxymaltose can reduce ESA requirements and improve iron status and hemoglobin in patients with ND-CKD.

**Methods:**

This prospective, single arm and single-center study included adult patients with ND-CKD (creatinine clearance ≤40 mL/min), hemoglobin 11–12 g/dL and iron deficiency (ferritin <100 μg/L or transferrin saturation <20%), who were regularly treated with oral iron and ESA during 6 months prior to inclusion. Study patients received an intravenous ferric carboxymaltose dose of 1,000 mg iron, followed by a 6-months ESA/ ferric carboxymaltose maintenance regimen (target: hemoglobin 12 g/dL, transferrin saturation >20%). Outcome measures were ESA dose requirements during the observation period after initial ferric carboxymaltose treatment (primary endpoint); number of hospitalizations and transfusions, renal function before and after ferric carboxymaltose administration, number of adverse reactions (secondary endpoints). Hemoglobin, mean corpuscular volume, ferritin and transferrin saturation were measured monthly from baseline until end of study. Creatinine clearance, proteinuria, C-reactive protein, aspartate aminotransferase, alanine aminotransferase and alkaline phosphatase bimonthly from baseline until end of study.

**Results:**

Thirty patients were enrolled (age 70.1±11.4 years; mean±SD). Mean ESA consumption was significantly reduced by 83.2±10.9% (from 41,839±3,668 IU/patient to 6,879±4,271 IU/patient; p<0.01). Hemoglobin increased by 0.7±0.3 g/dL, ferritin by 196.0±38.7 μg/L and transferrin saturation by 5.3±2.9% (month 6 vs. baseline; all p<0.01). No ferric carboxymaltose-related adverse events were reported and no patient withdrew or required transfusions during the study.

**Conclusion:**

Among patients with ND-CKD and stable normal or borderline hemoglobin, switching from oral iron to intravenous ferric carboxymaltose was associated with significant improvements in hematological and iron parameters and a significant reduction in ESA dose requirements in this single-center pilot study.

**Trial Registration:**

ClinicalTrials.gov NCT02232906

## Background

Anemia and iron deficiency are common complications in patients with chronic kidney disease (CKD) and contribute to the burden of disease in both patients with end-stage renal disease as well as those not yet requiring dialysis [1–4]. Anemia and iron deficiency can be associated with symptoms such as fatigue and dyspnoea [1,3–5] and can increase the risk of mortality and cardiovascular complications in patients with CKD [2,6,7]. Iron deficiency is the most common and reversible cause of anemia in patients with CKD and can occur independent of impaired kidney function [5].

Epidemiological data suggest that 58–73% of patients with creatinine clearance (CrCl) <60 mL/min are iron-deficient [8]. Factors contributing to the development of iron deficiency in CKD are reduced dietary iron intake, chronic inflammatory processes, rapidly increased iron requirements during treatment with erythropoiesis-stimulating agents (ESA) and, mainly in the case of hemodialysis patients, chronic blood loss [2].

Overall, treatment options for anemia in CKD mainly comprise iron, ESA and red blood cell (RBC) transfusions; yet guidelines recommend resolution of correctable causes of anemia such as iron deficiency before initiation of ESA and avoidance of RBC transfusions [5]. In line with clinical studies that evaluated different intravenous (i.v.) iron preparations in patients with hemodialysis-dependent (HD)-CKD and demonstrated a reduction of ESA dose requirements [9–17], i.v. iron therapy has become an integral part of anemia management in HD-CKD [5]. In patients with non-dialysis-dependent (ND)-CKD, several clinical studies and a meta-analysis have shown improvements in hemoglobin (Hb) and/or iron status in response to i.v. iron [18–25] and suggest that i.v. iron treatment may prevent or delay the need for ESA initiation [21–23,26]. However, there is currently no general consensus whether patients with ND-CKD and iron deficiency anemia should receive i.v. or oral iron as first-line therapy. The *Kidney Disease*: *Improving Global Outcomes* (KDIGO) anemia work group suggests a trial of i.v. iron for all adult anemic patients with CKD and iron deficiency (defined as serum ferritin ≤500 μg/L and transferrin saturation [TSAT] ≤30%) but states that in patients with ND-CKD, a trial of oral iron (1–3 months) can be used as an alternative [5].

In general, oral iron is often preferred for its convenience and low cost; however, its feasibility is often limited by gastrointestinal side effects, poor adherence to therapy, poor absorption, and low efficacy [27]. Notably, a meta-analysis of six clinical studies comparing the efficacy of i.v. and oral iron treatments in patients with ND-CKD showed statistically significantly better outcomes in Hb, serum ferritin and TSAT in i.v. iron-treated patients [25]. Also a recently published randomized controlled study comparing i.v. ferric carboxymaltose (FCM) and oral ferrous sulfate in patients with ND-CKD showed significantly better hematological outcomes and a delay in the need for other or additional anemia treatments in the i.v. iron compared to the oral iron arm [26].

The study presented here investigated whether a switch from oral iron to i.v. FCM could reduce ESA dose requirements and related costs in adult patients with ND-CKD who were on a stable ESA/oral iron schedule for 6 months prior to enrolment. Furthermore, effects on Hb levels and iron status were evaluated.

## Methods

The protocol for this trial and supporting TREND checklist are available as supporting information; see S1 TREND Checklist and S1 Protocol.

### Study design, patients and treatment

The study was designed as a 6-months prospective single arm study at the Department of Nephrology, Hospital Alemán, Buenos Aires, Argentina, complied with the Declaration of Helsinki and was authorized by the institutional review board (full name: “Committee on Education and Research institutions”) on 20^th^ December 2010 (S1 Protocol). Patient recruitment and follow-up took place from March 2011 to September 2013. All participating patients signed an informed consent form. The trial was registered at ClinicalTrials.gov (NCT02232906) after completion of the study. Early registration of the study has been overlooked since it was originally planned as a pilot study and it was not certain whether the study could be conducted. When the lack of registration was recognized, the study was registered retrospectively. The authors confirm that all ongoing and related trials for this drug at their institution are registered.

Eligible patients were >18 years of age with a measured CrCl ≤40 mL/min, Hb 11–12 g/dL, serum ferritin <100 μg/L or TSAT <20%, and had to receive monthly treatment with ESA and oral iron according to the institutional standard protocol for at least six months before enrolment in the study. Patients were excluded from the study if they had another obvious cause of acute or chronic anemia, or if they were expected to require hemodialysis within the next six months. Additional exclusion criteria were short life expectancy (<1 year), pregnancy, decompensated heart failure, history of allergic reactions to iron preparations and/or anaphylaxis from any cause, requirement of blood transfusions, chronic decompensated mental disorder or dementia.

**Fig 1:**
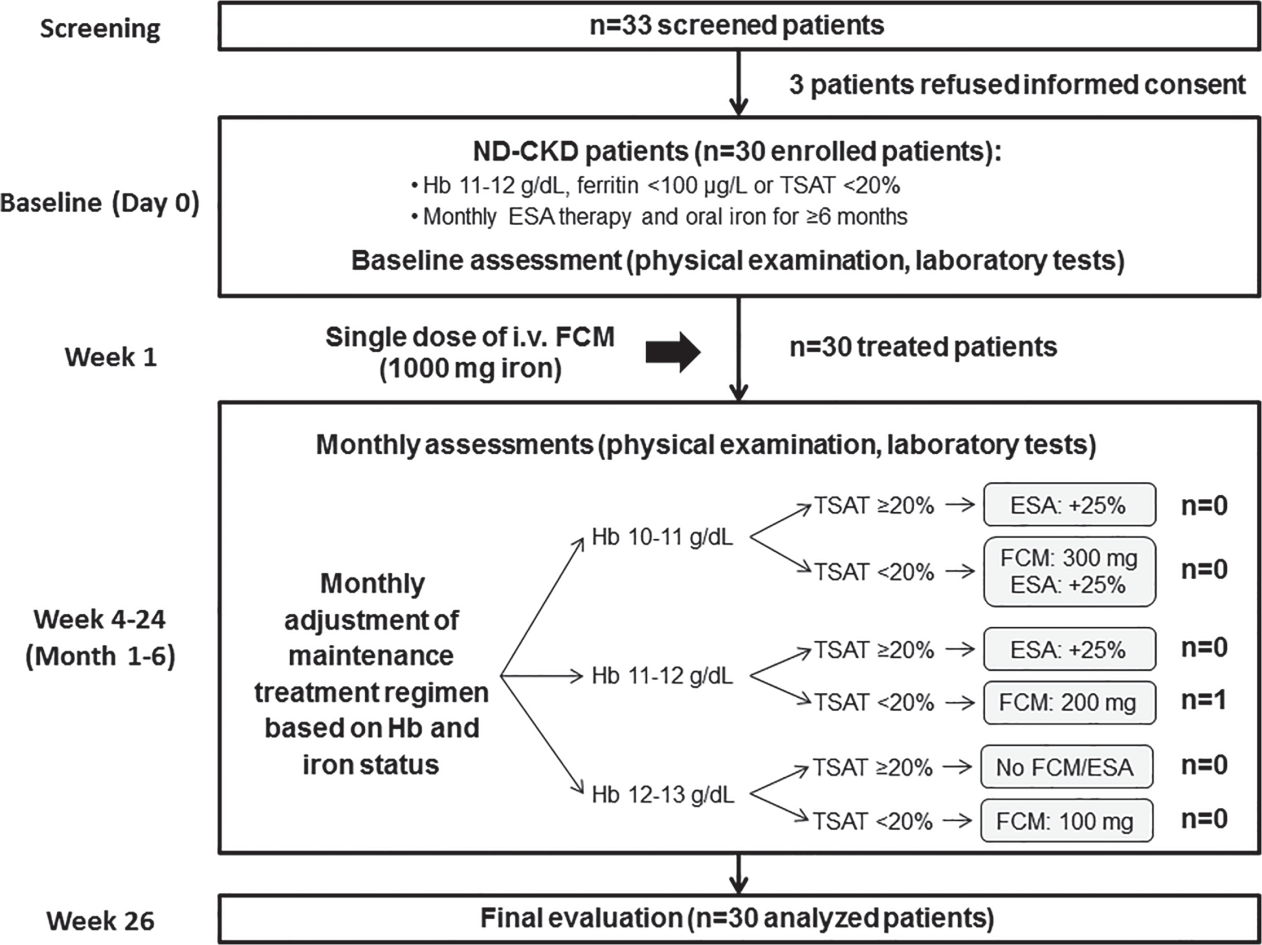
Study design, algorithm for maintenance therapy and CONSORT flow chart. After an initial dose of FCM (1,000 mg iron), patients were followed-up monthly and treated as necessary with a maintenance regimen that was based on a patient’s Hb level and TSAT. Abbreviations: ESA, erythropoiesis-stimulating agent; FCM, ferric carboxymaltose; Hb, hemoglobin; ND-CKD, non-dialysis chronic kidney disease; TSAT, transferrin saturation.

Enrolled patients underwent an initial physical examination and assessment of hematology parameters (day 0). In week 1 of the study, patients received a single i.v. FCM (Ferinject, Vifor Pharma, Switzerland) dose of 1,000 mg iron that was administered by a nurse under the supervision of the responsible physician in the outpatient setting of the Nephrology department. Thereafter, FCM and ESA doses to maintain Hb at 12 g/dL and TSAT above 20% (Fig 1) were adjusted based on monthly assessments of Hb and TSAT. Different brands of epoetin were used as ESA. The study duration was six months.

### Evaluation of outcomes

The primary endpoint was the ESA dose requirements during the observation period after the initial i.v. FCM treatment. Secondary endpoints were the number of hospitalizations and transfusions, renal function before and after FCM administration and the number of adverse reactions.

Hemoglobin, mean corpuscular volume (MCV), serum ferritin and TSAT as well as tolerability and adverse effects of FCM were assessed at baseline and monthly until end of study. Creatinine clearance, proteinuria, high sensitivity C-reactive protein (hs-CRP), aspartate aminotransferase (AST), alanine aminotransferase (ALT) and alkaline phosphatase (ALP) were assessed at baseline and then bimonthly until end of study.

### Statistical analysis

All statistical analyses were processed through GraphPad Prism version 6.0 (GraphPad Software, Inc. San Diego, CA, USA). Patient demographics as well as clinical and laboratory characteristics are presented by descriptive statistics. Gaussian or non-Gaussian distribution of every variable was assessed by Kolmogorov-Smirnov test. Repeated measures of variables with a Gaussian distribution were evaluated using repeated measures of ANOVA test and those with non-Gaussian distribution were evaluated with the Friedman Test (Nonparametric Repeated Measures ANOVA). Values are expressed as mean ± standard deviation (SD) and a p-value <0.05 was considered significant. Due to the exploratory purpose of this study, no standard sample size calculation was performed.

## Results

### Patient population

Thirty consecutive patients who fulfilled the inclusion and exclusion criteria were selected from the hospital’s CKD database and enrolled in the study (33 screened and eligible, 3 refused informed consent). The mean age of the study population was 70.1 ± 11.4 years and there was a comparable number of male (n = 16) and female (n = 14) patients (Table 1). All patients completed the study and were included in the final analysis.

**Table 1:** Baseline patient demographics.

	Mean ± SD
**Age (years)**	70.1 ± 11.4
**Gender, male/female n (%)**	16 (53.3%) / 14 (46.7%)
**Weight (kg)**	74.4 ± 9.5
**BMI (kg/m^2^)**	26.7 ± 2.9

BMI: body mass index; SD: standard deviation

### ESA and FCM dose requirements

**Fig 2:**
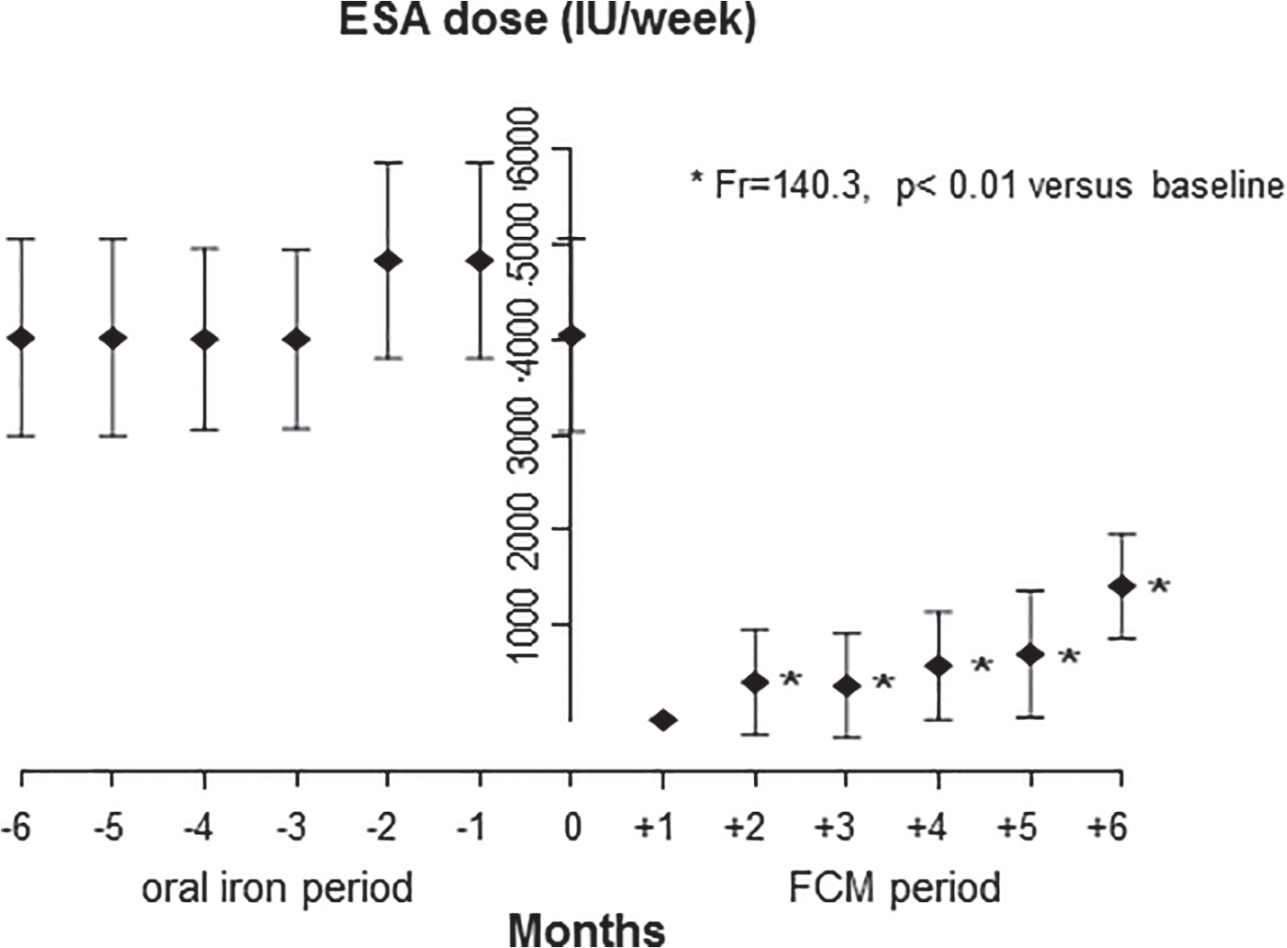
Mean monthly ESA dose requirements prior (months -6 to -1) and during the study (months +1 to +6).

The required ESA doses were significantly lower at all evaluated follow-up visits after the switch from oral iron to i.v. FCM (p<0.01 vs. baseline; Fig 2). At the first follow-up visit (month 1), no patient required ESA administration. Cumulative ESA consumption per patient over the entire 6-months study period after initial iron treatment was reduced by 83.2% ± 10.9%, with an average monthly requirement of 6,879 ± 4,271 IU/patient during the FCM period compared to 41,839 ± 3,668 IU/patient during the oral iron period (p<0.01). When considering only those 5 months of the FCM period in which ESA treatment was given, the average monthly ESA dose of 8,254 IU/patient, was 80.3% lower than the monthly dose during the oral iron period.

After the initial FCM dose of 1,000 mg iron, only one patient required an additional dose of FCM as per maintenance treatment regimen (200 mg iron, in month 5). In all other patients, TSAT remained ≥20% during the entire follow-up period.

### Hematological, biochemical and clinical variables

**Fig 3:**
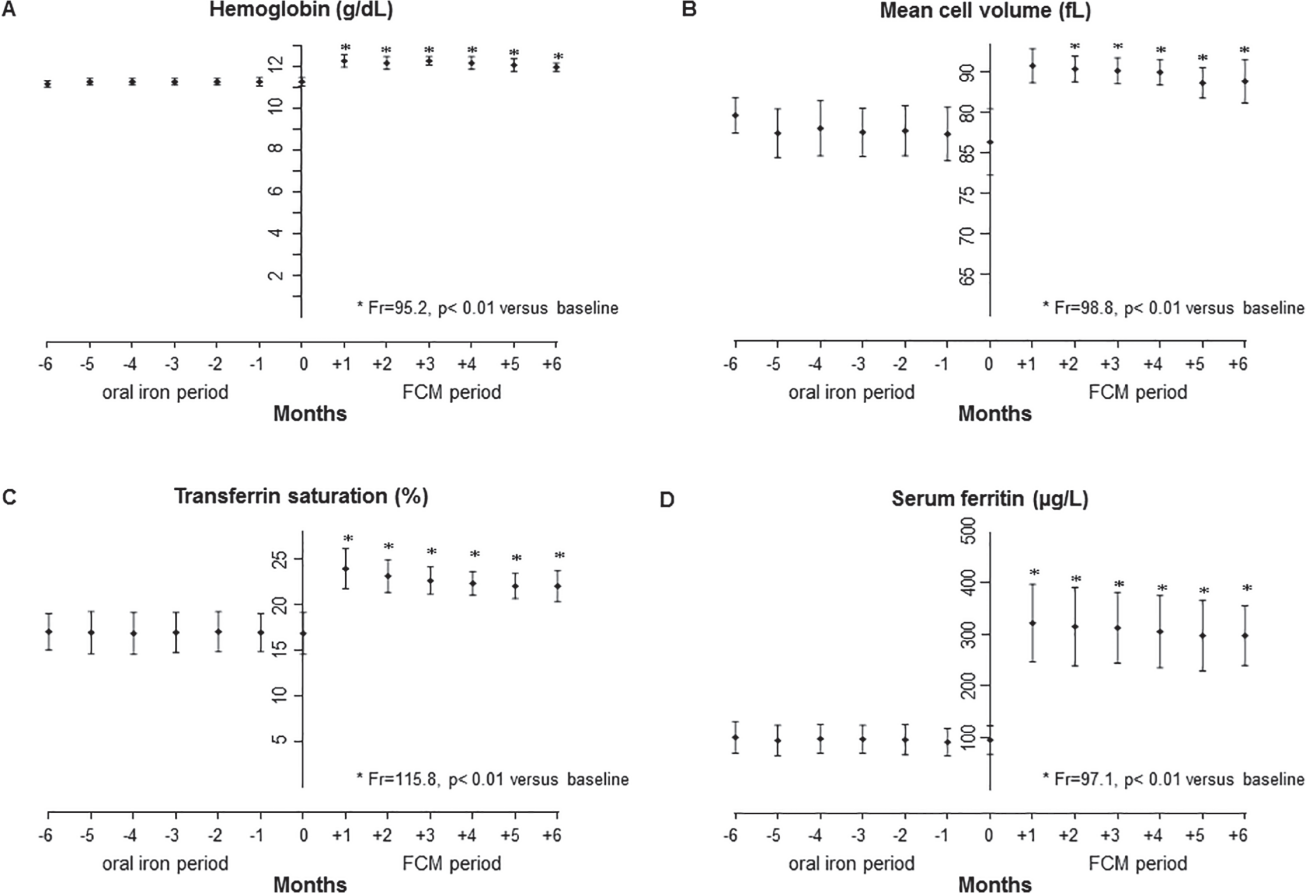
Levels of hematological and iron status variables. Mean monthly levels of (A) hemoglobin, (B) mean cell volume, (C) transferrin saturation, and (D) serum ferritin prior (months -6 to -1) and during the study (months +1 to +6).

Values of laboratory and clinical variables before (oral iron period) and after FCM administration were compared. Hematological variables (Hb, MCV) and iron status (serum ferritin, TSAT) significantly improved in patients after the initial FCM infusion (month 1) and subsequently remained at stable levels during the maintenance regimen, with mean values being higher at all follow-up visits (p<0.01 vs. baseline; Fig 3A–3D). At the end of the study, mean Hb was increased by 0.7 ± 0.3 g/dL, MCV by 7.5 ± 3.6 fL g/dL, serum ferritin by 196.0 ± 38.7 μg/L and TSAT by 5.3 ± 2.9% (month 6 vs. baseline, Table 2).

**Table 2:** Laboratory and clinical variables at baseline and end of study.

	Baseline (mean ± SD)	Month 6 (mean ± SD)
**Hb (g/dL)**	11.3 ± 0.2	12.0 ± 0.2*
**Serum ferritin (μg/L)**	95.6 ± 27.4	291.6 ± 27.4*
**TSAT (%)**	16.8 ± 2.3	22.1 ± 1.8*
**MCV (fL)**	81.3 ± 4.1	88.8 ± 2.9*
**sCr (mg/dL)**	4.4 ± 0.6	4.2 ± 0.7
**CrCl (mL/min)**	23.5 ± 6.0	24.5 ± 4.1
**Proteinuria (g/day)**	0.5 ± 0.6	0.6 ± 0.6
**hs-CRP (mg/L)**	6.0 ± 1.7	5.4 ± 1.6
**AST (IU/L)**	58.7 ± 8.6	60.1 ± 8.3
**ALT (IU/L)**	52.3 ± 9.1	53.9 ± 8.3
**ALP (IU/L)**	85.2 ± 6.5	86.9 ± 6.6
**Heart rate (beats/min)**	74.6 ± 10.3	73.4 ± 9.6
**Respiratory rate (breaths/min)**	17.1 ± 1.0	16.5 ± 1.3
**Systolic blood pressure (mm Hg)**	136.8 ± 9.5	135.3 ± 7.0
**Diastolic blood pressure (mm Hg)**	76.3 ± 9.1	76.0 ± 8.9

*p<0.01 vs. baseline.

SD: standard deviation; Hb: hemoglobin; TSAT: transferrin saturation; MCV: mean corpuscular volume; sCr: serum creatinine; CrCl: creatinine clearance; hs-CRP: high-sensitivity C-reactive protein; AST: aspartate aminotransferase; ALT: alanine aminotransferase; ALP: alkaline phosphatase

There were no significant changes in liver enzymes (AST, ALT, ALP), markers of renal function (CrCl and proteinuria) or inflammatory status (hs-CRP) (Table 2). Tested clinical parameters (heart and respiratory rate, systolic and diastolic blood pressure) were similar at baseline and at end of study (Table 2).

### Tolerability

During the FCM period (months 1–6), no drug-related adverse events were reported and no RBC transfusions were required. Renal function as measured by CrCl and proteinuria remained stable throughout the study. One patient was hospitalized due to dehydration.

During the oral iron period (months -6 to -1), a total of 27 drug-related adverse events were reported in 21 patients: diarrhea (3), constipation (11), nausea (5), upper abdominal discomfort (4), and metallic taste (4). Two patients were hospitalized due to unrelated respiratory tract infections.

### Economic impact

Cumulative drug acquisition costs during the 6-months study period (FCM/ESA) were US$ 3,070 per patient compared to US$ 17,670 per patient during the oral iron period (months -6 to -1), comprising savings of US$ 14,600 per patient or 82.6%.

## Discussion

The study reported herein evaluated whether a trial of oral iron before initiation of i.v. iron for the treatment of anemia in patients with ND-CKD, as recommended by the KDIGO guidelines [5], can be considered as optimal treatment in terms of minimal use of ESA (primary endpoint), Hb stabilization and iron repletion. In the investigated cohort of patients with ND-CKD who had stable Hb levels of 11–12 g/dL but were iron-deficient despite oral iron treatment for at least six months, a switch from oral iron to i.v. FCM significantly reduced ESA dose requirements, increased Hb levels and improved iron status. FCM administration was well tolerated and no drug-related adverse events were reported in the FCM treatment period. No RBC transfusions were required throughout the study. Notably, a single FCM dose of 1,000 mg iron was sufficient to maintain all but one patient iron-replete without additional iron treatment for the entire 6-months study period.

In patients with HD-CKD, i.v. iron treatment is accepted as standard of care [5] and several studies have demonstrated significant reductions in ESA dose requirements in stable HD patients [9–12,15,17], mixed populations of stable and new HD patients [13,14] and HD patients who were intolerant or unresponsive to oral iron [16]. Similar to our study in patients with ND-CKD, a study in stable HD patients also showed a 46.4% lower ESA dose requirement (p<0.05) in patients who were switched to i.v. iron compared to those who were continued on oral iron therapy [12].

In contrast to recommendations for patients with HD-CKD, anemia treatment guidelines recommend a trial of oral iron before i.v. iron treatment in patients with ND-CKD [5] although a meta-analysis of six studies (treatment duration 22 days to 6 months) showed significantly greater levels of Hb and serum ferritin in i.v. iron compared to oral iron-treated patients [25]. The results of this meta-analysis were recently confirmed by a large, prospective randomized study comparing i.v. FCM targeting a serum ferritin of 400–600 μg/L and oral iron (12 months treatment duration) in ND-CKD patients with anemia and iron deficiency (FIND-CKD) [26]. Furthermore, 6.5% of oral iron-treated patients in that study were switched to another iron treatment whereas a delayed and/or lower need for other anemia treatments was observed among FCM-treated patients. The potential effects of i.v. iron therapy beyond Hb and iron status and the question whether a trial of oral iron before initiation of i.v. iron is indeed the optimal approach for anemic patients with ND-CKD are largely unexplored.

Notably, all patients that were screened for the study reported here were iron-deficient although only patients who had achieved reasonable Hb levels (11–12 g/dL) with oral iron and ESA were considered for enrollment. In addition to the expected resolution of iron deficiency (increase of mean TSAT from below to above 20%), the switch from oral iron to i.v. FCM also resulted in achievement of significantly higher Hb levels and lower ESA dose requirements at all time points during the 6-months study period (Figs 2A and 3). These findings support the assumption that oral iron provides only suboptimal results even in patients considered responsive to oral iron. By this means the results of this study provide complementary evidence to the findings of other studies in patients who were unresponsive to oral iron [21] or who were not receiving ESA [19].

A 6-months study of i.v. iron sucrose treatment in patients with ND-CKD who were unresponsive to oral iron showed statistically significant Hb and hematocrit responses, and based on these results the authors suggested to increase serum ferritin levels and TSAT before initiating ESA treatment [21]. Another study that was one-year in duration evaluated i.v. iron sucrose without additional ESA and showed significant improvements in mean Hb and TSAT from month three onwards and maintenance of mean Hb and TSAT around 11.0 g/dL and 30%, respectively, until the end of the study [19].

The ESA dose reductions observed after switching patients with ND-CKD from oral iron to i.v. FCM are in line with those consistently reported for patients with HD-CKD (12% to 49%) and even exceed them substantially in magnitude [9–12,15,17]. Whether the slight increase in ESA dose requirements at the end of the 6-months study period reflects hyporesponsiveness to ESA due to recurring iron deficiency and could have been avoided by a less stringent threshold for the provision of FCM maintenance treatment (e.g. TSAT ≤30% and serum ferritin ≤500 μg/L according to KDIGO guidelines [5] rather than TSAT <20% as per study protocol) needs further investigation.

Since ESAs are the main drivers of anemia treatment costs, the reduction of ESA dose requirements in our study also reduced the cumulative acquisition costs of anemia drugs (ESA and iron) by more than 80% compared to the period when patients received oral iron. Calculations were based on health care costs in Argentina but similar savings may be possible in other countries. Notably, a more detailed pharmacoeconomic analysis would need to include the cost for drug administration such as nursing time and infusion sets. Apart from cost savings in patients with HD-CKD [9,13,16,28], an economic advantage with the use of i.v. iron has also been shown in oncology when anemic cancer patients were treated with a combination of ESA and i.v. iron sucrose compared with ESA alone [29,30]. In addition, an economic comparison of different i.v. iron formulations (FCM, iron sucrose, iron dextran and ferric gluconate) for iron therapy in cancer patients estimated that FCM had the lowest administration costs per 1,000 mg iron (even when FCM is given in two infusions of 500 mg iron each) [31].

Apart from concerns about ESA-related treatment costs, large randomized, controlled clinical trials in patients with ND-CKD have raised safety concerns associated with high-dose ESA therapy and aggressive Hb targets [32–34]. Treatment regimens utilizing ESA doses to achieve high target Hb levels ≥13.0 g/dL showed a higher event rate for a composite endpoint including death and hospitalization for chronic heart failure (CHOIR) [34], a higher number of patients requiring dialysis (CREATE) [32] and a higher rate of fatal or nonfatal stroke (TREAT) [33] compared to the respective treatment arm aiming for lower target Hb levels. Accordingly, options to minimize ESA dosing in the treatment of anemic patients with CKD are desired and should be considered in practice.

The absence of drug-related adverse events during the FCM period in the study reported here confirms the good tolerability of i.v. FCM in patients with ND-CKD as shown in several studies and patient populations. In contrast to the meta-analysis of randomized, controlled studies that suggested no difference in adverse events between oral and i.v. iron-treated patients with HD- or ND-CKD [25], the sequential comparison reported here showed a high rate of oral iron-related, mainly gastrointestinal, adverse events (27 events in 21 of 30 patients during the oral iron period), compared to no drug-related adverse events during the i.v. FCM period.

Notably, the benefits of i.v. iron treatment are not limited to anemic patients and not restricted to improved erythropoiesis as has been shown by studies in other therapeutic areas. In patients with chronic heart failure, a frequent comorbidity of CKD, i.v. iron therapy with FCM significantly improved disease symptoms, quality of life and functional capacity independent of the anemia status [35,36]. Improved iron management may also help in the prevention of thromboembolic events since a study in patients with inflammatory bowel disease revealed a mechanistic link between iron deficiency and secondary thrombocytosis [37]. In these patients, i.v. FCM reduced secondary thrombocytosis, platelet aggregation and P-selectin expression [38]. Also fatigue, the common symptom of iron deficiency and anemia, has been significantly reduced with a single FCM dose in a placebo-controlled study in iron-deficient women with normal or borderline Hb [39].

FCM has been chosen for this study since it is a stable iron carbohydrate complex that can be administered at a higher single dose per visit and over a shorter infusion time (20 mg iron/kg body weight over 15 minutes unless local labels set different limits) than other compounds such as ferric gluconate or iron sucrose [40,41]. Furthermore, FCM is a dextran-free i.v. iron preparation that has demonstrated a positive benefit/risk profile in many acute and chronic conditions [41].

Generalizability of the data is limited by the single-arm, single-center design and a relatively small patient number. Accordingly, validation of the results in a controlled study and larger patient sample from different centers is warranted.

## Conclusion

In conclusion, a single i.v. FCM dose of 1,000 mg iron was effective in reducing monthly ESA dose requirements and associated treatment costs, maintaining the recommended target Hb level, and increasing the iron parameters to target levels in patients with ND-CKD who had 6 months oral iron treatment before switching to FCM. Future controlled studies of i.v. iron in patients with ND-CKD to confirm the observed beneficial effects are warranted and should also consider the evaluation of potential non-hematological outcomes such as changes in fatigue, quality of life and exercise capacity.

## Supporting Information

S1 TREND Checklist TREND Checklist. (PDF)

S1 Protocol Trial protocol. (PDF)
