# Supplementary material for: Switching Patients with Non-Dialysis Chronic Kidney Disease from Oral Iron to Intravenous Ferric Carboxymaltose: Effects on Erythropoiesis-Stimulating Agent Requirements, Costs, Hemoglobin and Iron Status
Source: PLoS One. 2015 Apr 30;10(4):e0125528. doi: 10.1371/journal.pone.0125528 (PMC4415953; doi:10.1371/journal.pone.0125528)
Supplement: S1 Protocol — (PDF) [file pone.0125528.s002.pdf]

Jorge E. Toblli, MD; PhD; FASN.
Professor of Medicine University of Buenos Aires
Chief of Nephrology.
Hospital Alemán, Av. Pueyrredon 1640
Buenos Aires (1118)- Argentina
Fax: 54 11 4805-6087
Phone: 54 11 4 827- 7000
Mobile Phone: 54 9 11 53 17 04 15
E-mail: jorgetoblli@fibertel.com.ar

PILOT OPEN LABEL STUDY TO EVALUATE INTRAVENOUS FERRIC CARBOXYMALTOSE (FERINJECT ®) IN PATIENTS WITH CKD (pre-dialysis) WITH ANEMIA TREATED WITH EPO AND ORAL IRON IN BUENOS AIRES-ARGENTINA.

<u>Principal Investigator</u>

Professor Jorge E. Toblli, MD, PhD., FASN.

October 11, 2010

Jorge E. Toblli, MD; PhD; FASN.
Professor of Medicine University of Buenos Aires
Chief of Nephrology.
Hospital Alemán, Av. Pueyrredon 1640
Buenos Aires (1118)- Argentina
Fax: 54 11 4805-6087
Phone: 54 11 4 827- 7000
Mobile Phone: 54 9 11 53 17 04 15
E-mail: jorgetoblli@fibertel.com.ar

Intravenous FERRIC CARBOXYMALTOSE (FERINJECT ®) in CKD patients (pre-dialysis) with anemia treated with EPO and oral iron. Optimization of EPO cost.

Rational

Iron is an essential element that is crucial to all major metabolic pathways. Among others, it plays a central role in the proteins that transport oxygen in the blood and muscles, i.e. haemoglobin and myoglobin, respectively. The metabolism of iron is highly regulated with efficient recycling of iron but no active excretion of iron. Homeostasis is typically maintained at the level of intestinal absorption; however, iron deficiency may arise from blood loss, impaired uptake, dietary deficiency and developmental demands such as pregnancy. Iron deficiency is one of the most common nutrient deficiencies affecting an estimated two billion people worldwide (WHO health report 2002). If untreated, iron deficiency may lead to iron deficiency anaemia, whereas anaemia affects almost 25% of the world's population. Iron deficiency anaemia has been linked to impaired physical and mental development, fatigue, weakened immunity, poor work performance and a decreased quality of life.

Treatment ultimately involves identifying the underlying cause of the iron deficiency and replenishing the iron stores. Currently, oral supplementation is the first-line treatment though a significant number of patients do not tolerate or do not respond to oral iron therapy. In some studies, as many as 40% of patients reported side effects secondary to oral iron intake. Reported side effects usually occur within an hour after ingestion and include nausea, epigastric pain, vomiting, diarrhoea or constipation. Further limitations of oral iron treatment are impaired absorption, prolonged iron store repletion times and, particularly with concomitant erythropoiesis-stimulating agent (ESA) treatment, increased iron demands that cannot be matched by oral iron supplementation. Accordingly, a significant number of patients discontinue oral therapy despite substantial iron deficiency and require IV iron as an alternative approach.
Intravenous iron therapy is suitable in the following clinical situations:
a) intolerance to oral iron preparations; b) severe iron deficiency where a rapid therapeutic effect is needed; c) functional iron deficiency where the iron demand for haemoglobin synthesis exceeds the amount that can be mobilised from filled iron stores, e.g. in anaemia of inflammation or while using ESAs d) iron deficiency, where oral iron therapy is insufficient because of chronic blood loss; e) malabsorption of iron due to intestinal disorders; f) poor compliance to oral iron treatment: g) risk of drug-drug interactions between oral iron and concomitant medication.

Jorge E. Toblli, MD; PhD; FASN.
Professor of Medicine University of Buenos Aires
Chief of Nephrology.
Hospital Alemán, Av. Pueyrredon 1640
Buenos Aires (1118)- Argentina
Fax: 54 11 4805-6087
Phone: 54 11 4 827- 7000
Mobile Phone: 54 9 11 53 17 04 15
E-mail: jorgetoblli@fibertel.com.ar

Until recently, the most commonly used IV iron preparations comprised iron Dextran, iron gluconate, and iron sucrose. Although effective in replenishing iron stores, each of these formulations has distinct limitations. Iron Dextran is associated with reactivity to anti-Dextran antibodies that can induce hypersensitivity reactions and anaphylactic shock. On the other hand, less robust iron complexes such as iron gluconate and to lower extent iron sucrose release larger amounts of iron into the plasma. This can cause oxidative stress and loss of administered iron via renal excretion rather than incorporation into iron-binding proteins for utilisation in erythropoiesis. In an attempt to overcome these limitations, ferric Carboxymaltose has been developed as a novel IV iron repletion therapy.

Ferric Carboxymaltose represents a new generation of parenteral iron preparations that is currently approved in 17 EU countries (Austria, Czech Republic, Denmark, Estonia, Finland, Germany, Greece, Ireland, Latvia, Lithuania, The Netherlands, Poland, Portugal, Slovak Republic, Spain, Sweden and UK) as well as in Liechtenstein and Switzerland. During development the aim was to create a product that addresses the limitations of currently available IV iron products. In particular, the goal was to develop a formulation with the following properties:
1-dextran-free and thus not reactive with anti-dextran antibodies; 2-safe to be administered in a high dose; 3- administered over a shorter time than current products; 4- heat sterilisable; 5- pH-neutral and nearly isotonic.

Ferric Carboxymaltose is a Dextran-free iron complex consisting of a polynuclear iron (III) oxo-hydroxide core stabilised with a carbohydrate shell. The core of ferric Carboxymaltose resembles that of ferritin, the main intracellular iron storage protein. Ferric Carboxymaltose is taken up in the reticuloendothelial system of the liver, spleen, and bone marrow. The complex is degraded and the iron is made available for utilisation, i.e. incorporation in haemoglobin, or storage, i.e. incorporation in ferritin. As only very small amounts of unbound, labile iron are released into the circulation, the toxic effects of labile iron are avoided.

Ferric Carboxymaltose is a more stable complex than iron gluconate and iron sucrose. Consequently, it allows for administration of high single doses of iron. Despite the improved stability of iron sucrose compared to iron gluconate, both have to be administered at lower dosages than ferric Carboxymaltose, requiring more interventions for the patient and doctor.

Because of the efficient delivery of usable iron directly to the bone marrow, IV Ferric Carboxymaltose can play a vital role in treating anaemia of patient with CKD when used in combination with ESAs.

Jorge E. Toblli, MD; PhD; FASN.
Professor of Medicine University of Buenos Aires
Chief of Nephrology.
Hospital Alemán, Av. Pueyrredon 1640
Buenos Aires (1118)- Argentina
Fax: 54 11 4805-6087
Phone: 54 11 4 827- 7000
Mobile Phone: 54 9 11 53 17 04 15
E-mail: jorgetoblli@fibertel.com.ar

In agreement with this statement, several randomised studies in oncology patients have shown that ESAs were more effective when used with IV iron than with oral iron formulations alone. Furthermore, IV reduces the dose of ESAs required to achieve the target haemoglobin level. Once again, in patients with cancer-related anaemia, a Swedish study showed that total costs per patient over 16 weeks of treatment were reduced by 11% when a combined epoetin beta/iron sucrose protocol was used as compared with epoetin beta alone.

Although the pharmacoeconomic studies looking at cost-effectiveness were performed in Switzerland, similar savings may be possible in other countries (Latin America) due to the decrease in costs to the health-care system associated with fewer infusions and a sensible reduction in ESAs annual cost. Therefore, in the specific case of patients with anaemia and CKD, ferric Carboxymaltose may provide additional economic advantages.

OBJECTIVE

Primary:

- To determine possible reduction in ESA requirements after intravenous 1g Ferric Carboxymaltose (FERINJECT ®) administration in patients with CKD in pre-dialysis stage, who were under a monthly EPO treatment and continuous oral iron therapy during the previous six months, in order to maintain a Hb value according to international recommendations.

Secondary:

- To assess the number of hospitalizations pre (six months) and post (six months) intravenous 1g Ferric Carboxymaltose (FERINJECT ®) administration in patients with CKD in pre-dialysis stage.

- To record the need for transfusion pre (six months) and post (six months) intravenous 1g Ferric Carboxymaltose (FERINJECT ®) administration in patients with CKD in pre-dialysis stage.

- To evaluate renal function as measured by Creatinine clearance and proteinuria of 24 hours, pre (six months) and post (six months) intravenous 1g Ferric Carboxymaltose (FERINJECT ®) administration in patients with CKD in pre-dialysis stage.

- To analyze the number of adverse reactions and complications due to intravenous 1g Ferric Carboxymaltose (FERINJECT ®) administration in patients with CKD in pre-dialysis stage.

Jorge E. Toblli, MD; PhD; FASN.
Professor of Medicine University of Buenos Aires
Chief of Nephrology.
Hospital Alemán, Av. Pueyrredon 1640
Buenos Aires (1118)- Argentina
Fax: 54 11 4805-6087
Phone: 54 11 4 827- 7000
Mobile Phone: 54 9 11 53 17 04 15
E-mail: jorgetoblli@fibertel.com.ar

Inclusion Criteria

- Patients over 18 years with chronic kidney disease (Creatinine Clearance ≤ 40 ml / min), and Hb between 11-12 g / dl, serum ferritin <100 mg / dl, or TSAT <20, being treated with a regular (monthly) institution schedule for EPO together with oral iron during the last 6 months before inclusion.

Exclusion Criteria

- Patients with other obvious cause of acute anemia (gastrointestinal bleeding, urological or gynaecological), or other causes of chronic anemia (active malignancy, chronic infections).

- Patients expected to require HD in next 6 months.

- Patients with decompensate heart failure.

- History of allergic reactions to iron preparations and / or anaphylaxis from any cause.

- Patients requiring blood transfusion and blood products for another reason (planned major surgery).

- Pregnant patients.

- Patients with chronic decompensate mental disorders.

- Bedridden patients with dementia syndromes or unable to sign informed consent.

- Patients with a life expectancy of less than a year.

Jorge E. Toblli, MD; PhD; FASN.
Professor of Medicine University of Buenos Aires
Chief of Nephrology.
Hospital Alemán, Av. Pueyrredon 1640
Buenos Aires (1118)- Argentina
Fax: 54 11 4805-6087
Phone: 54 11 4 827- 7000
Mobile Phone: 54 9 11 53 17 04 15
E-mail: jorgetoblli@fibertel.com.ar

STUDY DESIGN

- Prospective study to 6 months with a single branch of patients who are treated with monthly EPO therapy together with oral iron for at least the last six months before the study and met inclusion criteria.

- From CKD database are randomly selected 30 patients. After signing an informed consent form in duplicate and with witness, duly authorized by the Committee on Education and Research institutions, all patients will receive intravenous 1g Ferric Carboxymaltose (FERINJECT ®) in one dose of 1000 mg on the recommendation of the product.

- On a monthly basis after intravenous 1g Ferric Carboxymaltose (FERINJECT ®) the administration, all patients will be controlled dosage of Hb by HemoCue, in order to dispense the EPO to maintain a baseline Hb level.

- Monthly checks will be carried to register: vital signs and weight control, and record of adverse reactions linked to 1g Ferric Carboxymaltose (FERINJECT ®) administration, registration HemoCue results.

- Before FERINJECT treatment and then every two months, all patients underwent blood control that includes: complete blood count haematimetric indexes, platelet count, serum iron, serum ferritin, TSAT, CRP, urea, creatinine, liver function tests).

- There will be collection of 24-hour urine to measure Creatinine Clearance and proteinuria at baseline and at the end of the study.

Jorge E. Toblli, MD; PhD; FASN.
Professor of Medicine University of Buenos Aires
Chief of Nephrology.
Hospital Alemán, Av. Pueyrredon 1640
Buenos Aires (1118)- Argentina
Fax: 54 11 4805-6087
Phone: 54 11 4 827- 7000
Mobile Phone: 54 9 11 53 17 04 15
E-mail: jorgetoblli@fibertel.com.ar

METHODS

This is a pilot, open label study in which the population will be formed by adult patients either gender, with CKD in pre-dialysis stage in Buenos Aires, Argentina, that fulfill the inclusion and exclusion criteria previously established.

Once signed the informed consent, the patients will be included in the study an initial check-up will be performed in order to evaluate clinical status and to assess haematology parameters in every patient.

After that the patients will receive intravenous Ferric Carboxymaltose (Ferinject ®) by the following therapeutic schedule:

Visit 2: IV FERINJECT ® 1000 mg in an infusion over 15-20 minutes.

Then, a monthly evaluation will be performed by six months.
During the time of the investigation there will be strict control of adverse effects in every patient and that will be recorded in the corresponding CRF.

Follow-up Phase:
In each check-up, measurements of the haematological parameters will be taken so to assess the efficacy tolerability and potential adverse effect of Ferric Carboxymaltose (Ferinject ®) in every studied patient.

Chronogram of visits
Initial Assessment (visit 1): It will be performed the day the patient enters the study once he/she has read and signed the Informed Consent regarding the participation in the study. In this assessment the following information will be collected:

a) Demographic and anthropometric data: City and country of residence, age, sex, weight, height, occupation, socioeconomic strata.
b) Medical History: Diagnosis associated.
c) Physical Examination (Vital signs: blood pressure, heart rate, breath rate, etc).
d) Blood samples for Hb, Hct. RBC, serum ferritin, transferrin saturation, creatinine clearance, and proteinuria.

Jorge E. Toblli, MD; PhD; FASN.
Professor of Medicine University of Buenos Aires
Chief of Nephrology.
Hospital Alemán, Av. Pueyrredon 1640
Buenos Aires (1118)- Argentina
Fax: 54 11 4805-6087
Phone: 54 11 4 827- 7000
Mobile Phone: 54 9 11 53 17 04 15
E-mail: jorgetoblli@fibertel.com.ar

Visit 2:
a) Anamnesis and Physical Examination (Vital signs: blood pressure, heart rate, breath rate, etc).
b) IV Ferinject ® 1000 mg administration.
c) Evaluation of tolerability and adverse effects.

Visit 3
a) Anamnesis and Physical Examination (Vital signs: blood pressure, heart rate, breath rate, etc).
b) Evaluation of tolerability and adverse effects.
c) Blood samples for Hb, Htc. RBC, serum ferritin, transferrin saturation, creatinine clearance and proteinuria.

Visit 4 to 8
a) Anamnesis and Physical Examination (Vital signs: blood pressure, heart rate, breath rate, etc).
b) Evaluation of tolerability and adverse effects.
c) Blood samples for Hb, Htc. RBC, serum ferritin, transferrin saturation, creatinine clearance and proteinuria.
d) Adjustment of medication according to the Hb and TSAT outcomes.

**Potential results post** FERINJECT ®		**Measure to follow**	
1- Hb 12-13 (g/dl)	TSAT (%) > 20	NO Ferinject	NO Epo
2- Hb 12-13 (g/dl)	TSAT (%) < 20	Ferinject 100mg	NO Epo
3- Hb 11-12 (g/dl)	TSAT (%) > 20	NO Ferinject	increase Epo 25%
4- Hb 11-12 (g/dl)	TSAT (%) < 20	Ferinject 200mg	NO Epo
5- Hb 10-11 (g/dl)	TSAT (%) > 20	NO Ferinject	increase Epo 25%
6- Hb 10-11 (g/dl)	TSAT (%) < 20	Ferinject 300mg	increase Epo 25%

Visit 9 (end of the study):
a) Final evaluation

Jorge E. Toblli, MD; PhD; FASN.
Professor of Medicine University of Buenos Aires
Chief of Nephrology.
Hospital Alemán, Av. Pueyrredon 1640
Buenos Aires (1118)- Argentina
Fax: 54 11 4805-6087
Phone: 54 11 4 827- 7000
Mobile Phone: 54 9 11 53 17 04 15
E-mail: jorgetoblli@fibertel.com.ar

Patient withdrawal

All patients of the population study may withdraw any time from the investigation by own will or by the investigator`s decision before the occurrence of any adverse effect or transitory disease that could alter the results of the study or that represents a contraindication for the administration of the medicine. The reasons for withdrawal will have to be documented.

Jorge E. Toblli, MD; PhD; FASN.
Professor of Medicine University of Buenos Aires
Chief of Nephrology.
Hospital Alemán, Av. Pueyrredon 1640
Buenos Aires (1118)- Argentina
Fax: 54 11 4805-6087
Phone: 54 11 4 827- 7000
Mobile Phone: 54 9 11 53 17 04 15
E-mail: jorgetoblli@fibertel.com.ar

FLOW CHART

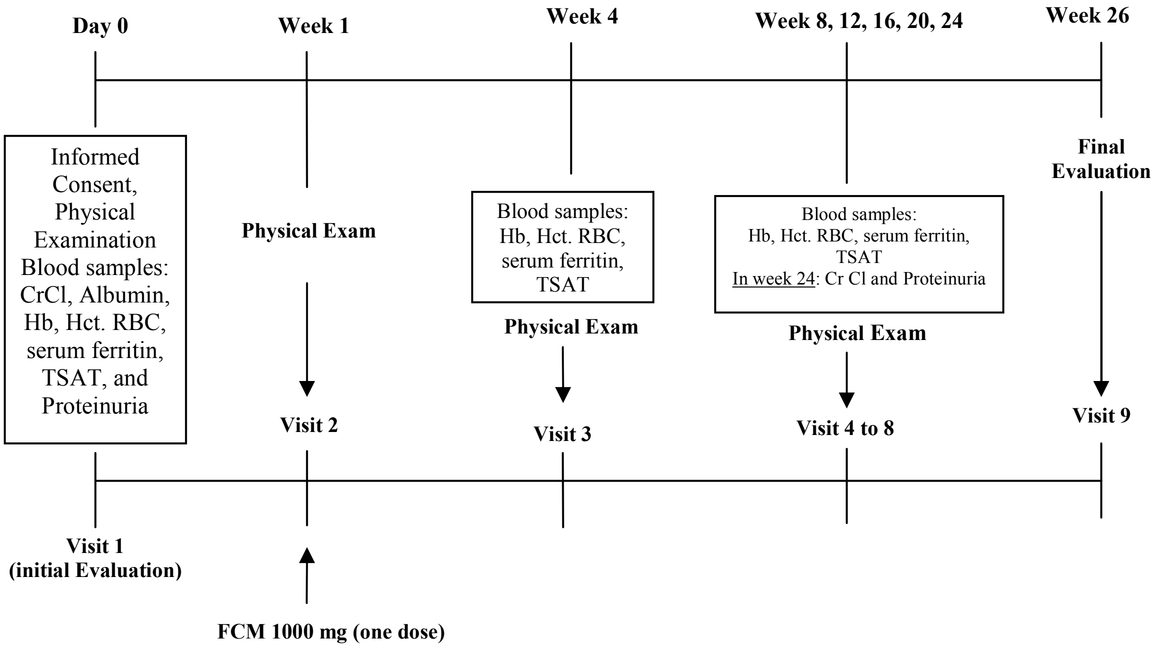

FCM= Ferric Carboxymaltose Ferinject ®)

Jorge E. Toblli, MD; PhD; FASN.
Professor of Medicine University of Buenos Aires
Chief of Nephrology.
Hospital Alemán, Av. Pueyrredon 1640
Buenos Aires (1118)- Argentina
Fax: 54 11 4805-6087
Phone: 54 11 4 827- 7000
Mobile Phone: 54 9 11 53 17 04 15
E-mail: jorgetoblli@fibertel.com.ar

POPULATION AND SAMPLE

Population
The reference population will be patient resident adults in Buenos Aires who are covered by any regime of social security, taking as population of study those attended in the hospital who fulfil the exclusion and inclusion criteria.

Study Sample
Thirty consecutive patients who fulfilled the inclusion and exclusion criteria

VARIABLES
1-Dependent Variables
- Heart rate
- Breath rate
- Blood pressure
- Severity of the Anemia: According to the classification established by the WHO.
- Hemoglobin: substance inside erythrocytes consisting of a protein that binds iron
- Hematocrit: Proportion of erythrocytes in plasma
- Mean Corpuscular Volume (MCV): cellular index that reflects erythropoiesis.
- Serum Ferritin
- Percentage of transferrin saturation
- Creatinine Clearance
- Proteinuria
- Amount of Erythropoietin.
- Number of transfusions
- Number of hospitalizations
- Death
- Cause of Death
- Adverse Events: Appearance of adverse reactions in the organism with the treatment administered. Among others: nausea, vomiting, headache, diarrhea, allergy and hypotension.

2-Independent variables
- CV Drugs (It includes Angiotensin Converting Enzyme Inhibitors and Antagonist of the Angiotensin II receptor type 1).

Jorge E. Toblli, MD; PhD; FASN.
Professor of Medicine University of Buenos Aires
Chief of Nephrology.
Hospital Alemán, Av. Pueyrredon 1640
Buenos Aires (1118)- Argentina
Fax: 54 11 4805-6087
Phone: 54 11 4 827- 7000
Mobile Phone: 54 9 11 53 17 04 15
E-mail: jorgetoblli@fibertel.com.ar

DATA COLLECT METHODOLOGY

The application of all forms will be done based on a primary source, i.e. the information will be taken by the investigators through direct evaluation of the patients by physical examination and the results from their blood and serum sample analysis.

- 1: Initial clinical and laboratory evaluation of the patient by means of direct observation and the transcription of the obtained results of laboratory as a point to begin. (Appendix 1)

- 2: Clinical and laboratory evaluation of the follow-up of the patient by means of direct observation and the transcription of the obtained results of laboratory during the different moments in the follow-up. (Appendix 1)

- 3: Record of symptoms and side effects. It will be record by the investigator in every visit (Appendix 1)

DATA COLLECT TECHNIQUES

Pertinent authorization formalities
The investigation project will be submitted to the ethics and investigation committees of the Hospital Alemán (Deutches Hospital) with the aim of obtaining the corresponding permissions for the accomplishment of the study.

Recruitment and training of the personnel
It will be supported by the principal and co-investigators, who will participate in the initial evaluations and in the follow-up of the patients. Before initiating the study, an instruction will be given as how to fill the forms.

Data sources and data collecting procedures
In order to collect the data, the direct observation through clinical examination and blood sample analysis and serum will be used. The registry of the data obtained will be done in the corresponding forms.

Jorge E. Toblli, MD; PhD; FASN.
Professor of Medicine University of Buenos Aires
Chief of Nephrology.
Hospital Alemán, Av. Pueyrredon 1640
Buenos Aires (1118)- Argentina
Fax: 54 11 4805-6087
Phone: 54 11 4 827- 7000
Mobile Phone: 54 9 11 53 17 04 15
E-mail: jorgetoblli@fibertel.com.ar

STATISTICS

All statistical analyses will be processed through GraphPad Prism version 4.0 (GraphPad Software, Inc. San Diego, CA). Initially, a descriptive statistic of the sociodemographic, clinical and laboratory characteristics will be performed of all the participant patients of the study, directed to describe patient population. Then, calculation of absolute frequencies, proportions, measures of central tendency and dispersion of the involved variables will be performed. When evaluating parameters with a Gaussian distribution, comparisons will be carried out using t-test. For those parameters with a non-Gaussian distribution, comparisons will be performed by non-parametric methods using the Wilcoxon rank sum test. Values will be expressed as mean ± SD, and $p<0.05$ will be considered significant.

ETHICAL ISSUES

The following issues will be considered:

A-Protection of the professional secret. The identification data of the participant patients will be replaced assigning a code to them at the time of entering the study

B-Informed Consent: Once obtained the reference population, there will be an invitation to participate in the study to the patients who fulfil the inclusion and exclusion criteria already mentioned. To those interested there will be an explanation of the study objectives and an Informed Consent will be given which includes information about the importance of the present investigation, the characteristics of the study and the benefits and potentials risks that they will be exposed when participating in the project. If the patient is illiterate, an acceptable legal representative will have to sign the informed consent.

C-During the accomplishment of the study, every 4 weeks, partial information will be taken to evaluate the treatment effectiveness and its safety allowing the detection of hazardous situations that can risk the health conditions of the participants in the study.

Deutsches Hospital

Jorge E. Toblli, MD; PhD; FASN.
Professor of Medicine University of Buenos Aires
Chief of Nephrology.
Hospital Alemán, Av. Pueyrredon 1640
Buenos Aires (1118)- Argentina
Fax: 54 11 4805-6087
Phone: 54 11 4 827- 7000
Mobile Phone: 54 9 11 53 17 04 15
E-mail: jorgetoblli@fibertel.com.ar

REFERENCES

Anker SD, Comin Colet J, Filippatos G, Willenheimer R, Dickstein K, Drexler H, Lüscher TF, Bart B, Banasiak W, Niegowska J, Kirwan BA, Mori C, von Eisenhart Rothe B, Pocock SJ, Poole-Wilson PA, Ponikowski P; FAIR-HF Trial Investigators. Ferric carboxymaltose in patients with heart failure and iron deficiency. N Engl J Med. 2009; 361(25):2436-2448.

Chandler G, Harchowal J, Macdougall IC. Intravenous iron sucrose: Establishing a safe dose. Am J Kidney Dis 2001;38: 988-991

Crichton RR., Danielson BG, Geisser P. Iron therapy with special emphasis on intravenous administration. 4th edition, Uni-Med, 2008

Geisser P, Baer M, Schaub E. Structure/ histotoxicity relationship of parental iron preparations. Arnzeim. Forsch./Drug Res 1992; 42:1439-1452

Geisser P The pharmacology and safety profile of ferric carboxymaltose (Ferinject®): structure/reactivity relationships of iron preparations. Port J Nephrol Hypert 2009; 23(1): 11-16

Hedenus M, Birgegård G. The role of iron supplementation during epoietin treatment for cancer-related anemia. Med Oncol 2009; 26 (1):105-15.

Hedenus M, Birgegård G, Näsman P, et al. Addition of intravenous iron to epoetin beta increases hemoglobin response and decreases epoetin dose requirement in anemic patients with lymphoprolifera-tive malignancies: a randomized multi-center study. Leukemia 2007; 21(4): 627-32

Hedenus M, Näsman P, Liwing J. Economic evaluation in Sweden of epoetin beta with intravenous iron supplementation in anaemic patients with lymphoproliferative malignancies not receiving chemotherapy. J Clin Pharm Ther 2008; 33 (4):365-74

Silverberg D.S., Wexler D., Blum M. et al. The Use of Subcutaneus Erythropoietin and Intravenous Iron for the Treatment of the Anemia of Severe, Resistant Congestive Heart Failure Improves Cardiac and Renal Function and Functional Cardiac Class, and Markedly Reduces Hospitalizations. J Am Coll Cardiol; 35 (7): 1737-1744

Silverberg D.S. Intravenous iron supplementation for the treatment of the anemia of moderate to severe chronic renal failure patients not receiving dialyses. Am J Kidney Dis 1996; 27(2):234-238.

Jorge E. Toblli, MD; PhD; FASN.
Professor of Medicine University of Buenos Aires
Chief of Nephrology.
Hospital Alemán, Av. Pueyrredon 1640
Buenos Aires (1118)- Argentina
Fax: 54 11 4805-6087
Phone: 54 11 4 827- 7000
Mobile Phone: 54 9 11 53 17 04 15
E-mail: jorgetoblli@fibertel.com.ar

Shord SS, Hamilton JM Jr, Cuellar S. Parenteral iron with erythropoiesis-stimulating agents for chemotherapy-induced anemia. J Oncol Pharm Pract 2008;14 (1):5-22

Toblli JE, Lombraña A, Duarte P, Di Gennaro F. Intravenous iron reduces NT-pro-brain natriuretic peptide in anemic patients with chronic heart failure and renal insufficiency. J Am Coll Cardiol. 2007; 50 (17):1657-1665

Toblli JE Cao G, Oliveri L, Angerosa M. Different toxicity of renal tissues between new and old original intravenous iron preparations in normal rats. Am J Kidney Dis 2008; 51: 535-712 (A92)

Toblli JE, Cao G, Oliveri L, Angerosa M. Evaluation of intravenous iron preparations on cardiovascular toxicity in normal rats. J Am Soc Nephrol 2008; 19: 918A

Toblli JE, Cao G, Olivieri L, Angerosa M. Comparison of the renal, cardiovascular and hepatic toxicity data of original intravenous iron compounds. Nephrol Dial Transplant. 2010 Aug 19. [Epub ahead of print] PMID: 20488820 [PubMed - as supplied by publisher]

Timelines

a. FPFV: 14/03/2011
b. LPFV: 09/11/2012
c. LPLV: 10/05/2013
d. FR: 08/07/2013
